# Circular RNA circ_IRAK3 contributes to tumor growth through upregulating KIF2A via adsorbing miR-603 in breast cancer

**DOI:** 10.1186/s12935-022-02497-y

**Published:** 2022-02-14

**Authors:** Fang Wang, Jingruo Li, Lin Li, Zhuo Chen, Nan Wang, Mingzhi Zhu, Hailong Mi, Youyi Xiong, Guangcheng Guo, Yuanting Gu

**Affiliations:** Department of Breast Surgery, The First Affiliated Hospital of Zhengzhou University, No. 1, Jianshe East Road, Erqi District, Zhengzhou, 450000 Henan China

**Keywords:** BC, circ_IRAK3, miR-603, KIF2A

## Abstract

**Background:**

Breast cancer (BC) threatens the health of women around the world. Researchers have proved that hsa_circ_0005505 (circ_IRAK3) facilitates BC cell invasion and migration, but the regulatory mechanisms of circ_IRAK3 in BC remain mostly unknown. We aim to explore a new mechanism by which circ_IRAK3 promotes BC progression.

**Methods:**

Levels of circ_IRAK3, microRNA (miR)-603, and kinesin family member 2A (KIF2A) mRNA in BC tissues and cells were examined by quantitative real-time polymerase chain reaction (qRT-PCR). The cell cycle progression, colony formation, and proliferation of BC cells were evaluated by flow cytometry, plate clone, or 3-(4,5-dimethylthiazol-2-yl)-2,5-diphenyltetrazoliumbromide (MTT) assays. The migration, invasion, and apoptosis of BC cells were determined by transwell or flow cytometry assays. Several protein levels were detected using western blotting. The targeting relationship between circ_IRAK3 or KIF2A and miR-603 was verified via dual-luciferase reporter assay. The role of circ_IRAK3 in vivo was verified by xenograft assay.

**Results:**

We observed higher levels of circ_IRAK3 in BC tissues and cell lines than their respective controls. Functional experiments presented that circ_IRAK3 silencing induced BC cell apoptosis, curbed cell proliferation, migration, and invasion in vitro, and decreased tumor growth in vivo. Mechanistically, circ_IRAK3 could modulate kinesin family member 2A (KIF2A) expression through acting as a microRNA (miR)-603 sponge. miR-603 silencing impaired the effects of circ_IRAK3 inhibition on the malignant behaviors of BC cells. Also, the repressive effects of miR-603 mimic on the malignant behaviors of BC cells were weakened by KIF2A overexpression.

**Conclusions:**

circ_IRAK3 exerted a promoting effect on BC progression by modulating the miR-603/KIF2A axis, providing a piece of novel evidence for circ_IRAK3 as a therapeutic target for BC.

**Supplementary Information:**

The online version contains supplementary material available at 10.1186/s12935-022-02497-y.

## Introduction

Breast cancer (BC) is a complex heterogeneous disease in women, accounting for about 25% of female cancer cases [1, 2]. The treatment of BC depends on the patient’s tumor characteristics and personal factors [3]. Early BC patients have a good prognosis, but metastatic and chemo-resistant BC patients have a poor prognosis [4]. At present, the mechanism related to the biological behaviors of BC is still unclear, so it is necessary to study new mechanisms to provide latent therapeutic targets [5].

Circular RNAs (circRNAs), a type of covalently closed circular RNAs, are produced from linear transcripts through back-splicing [6]. CircRNAs are resistant to the degradation of exonuclease RNase R because of their unique structures [7]. Mounting evidence has manifested that circRNAs exert important roles in some pathological and physiological processes [8]. In recent years, increased studies have indicated that circRNAs are implicated in the progression of BC. For instance, circRNA circ_ABCB10 [9], circRNA hsa_circ_0000515 [10], and circRNA circ_RAD18 [11] could accelerate the growth of BC. Nevertheless, the regulatory mechanism of circRNAs in BC is indistinct. Circular RNA circ_IRAK3 (circ_IRAK3, also termed as hsa_circ_0005505), located at chr12: 66597490-66622150, originates from the interleukin 1 receptor-associated kinase 3 (IRAK3) gene with a splice length of 754 nt. It was reported that circ_IRAK3 expression was elevated in hepatocellular carcinoma [12]. Also, the recurrence of BC was associated with the upregulation of circ_IRAK3 [13]. However, the molecular mechanisms by which circ_IRAK3 regulates BC progression are unclear.

MicroRNAs (miRs) can modulate gene expression by repression of mRNA translation or acceleration of mRNA degradation [14]. MiRs are also connected with the modulation of many biological processes and participate in the development of various diseases [15]. Recent researches have proved that circRNAs take part in the advancement of diverse cancers via functioning as miR sponges [16]. For example, circRNA circ_TLK1 contributed to renal cell carcinoma cell metastasis and proliferation via sponging miR-136-5p [17]. CircRNA circ_LPAR3 sponged miR-198 to accelerate tumor metastasis in esophageal cancer [18]. Through the prediction of the circBank database, we found that circ_IRAK3 may interact with miR-603, which plays a suppressive role in BC [19]. However, the relationship between circ_IRAK3 and miR-603 in BC is unclear. KIF2A, a member of the kinesin-13 family, belongs to M-type nonmotile microtubule depolymerase [20]. KIF2A has been reported to be upregulated in many tumor tissues [21, 22]. In addition, we discovered that KIF2A may be a target of miR-603 using the miRDB database (http://mirdb.org/). However, the targeting relationship between KIF2A and miR-603 has not been reported yet.

In the current study, we proved that circ_IRAK3 was overexpressed in BC tissues and samples. Moreover, circ_IRAK3 silencing reduced BC cell growth in vitro and in vivo. Mechanistically, circ_IRAK3 could adsorb miR-603 to regulate KIF2A expression. Thus, the study uncovered that circ_IRAK3 might drive BC progression via KIF2A through sponging miR-603, which provided a novel molecular mechanism related to BC advancement. The flowchart of our study was illustrated in Additional file 1: Fig. S1.

## Materials and methods

### Patient-derived samples

The research was authorized by the Ethics Committee of the First Affiliated Hospital of Zhengzhou University. Forty seven paired tumor tissues and adjacent non-cancerous tissues were collected from BC patients who underwent surgery at the First Affiliated Hospital of Zhengzhou University between April 2017 and December 2019. All participants signed informed consent and did not received radiotherapy, chemotherapy, or other anti-tumor treatment.

### Cell culture

BC cells (HCC70 and MDA-MB-231) and human mammary gland epithelial cells (MCF-10A) were bought from the American Type Culture Collection (Manassas, VA, USA). These cells were cultured in mammary epithelial cell growth medium (MEGM) BulletKit (Lonza, Basel, Switzerland) (MCF-10A cells) or Roswell Park Memorial Institute-1640 medium (Sigma, St Louis, MO, USA) (HCC70 and MDA-MB-231) supplemented with 10% fetal bovine serum (FBS) (Sigma) and 1% streptomycin/penicillin (Solarbio, Beijing, China) and maintained at 37 °C in a moist atmosphere with 5% CO_2_.

### Cell transfection

Transient transfection was performed using Lipofectamine 3000 reagent (Thermo Fisher Scientific, Waltham, MA, USA). Small interference (si) RNA against circ_IRAK3 (si-circ_IRAK3: 5′-TCTCTCTGCTTGATATACTGC-3′) and its negative control (si-NC: 5′-TTCTCCGAACGTGTCACGTAA-3′) were synthesized by Geneseed (Guangzhou, China). MiR-603 mimic (miR-603) and its negative control (miR-NC), as well as miR-603 inhibitor (anti-miR-603) and its negative control (anti-miR-NC), were purchased from GenePharma (Shanghai, China). The full-length sequence of KIF2A was cloned into empty pcDNA3.1 vector (pcDNA) (Life Technologies, Grand Island, NY, USA) for pcDNA3.1-KIF2A (KIF2A) generation. Lentiviral vectors carrying sh-circ_IRAK3 (sh-circ_IRAK3: 5′-CCGGTCTCTCTGCTTGATATACTGCCTCGAGGCAGTATATCAAGCAGAGAGATTTTTTG-3′) and its negative control (sh-NC: 5′-CCGGTTCTCCGAACGTGTCACGTAACTCGAGTTACGTGACACGTTCGGAGAATTTTTG-3′) were constructed by Geneseed. MDA-MB-231 cells stably knockdown circ_IRAK3 were constructed by infecting with lentiviral particles and selecting with puromycin (Solarbio).

### Quantitative real-time polymerase chain reaction (qRT-PCR)

Total RNA (tissue samples and cells) was extracted with the miRNeasy Mini Kit (Qiagen, San Diego, CA, USA). Nuclear and cytoplasmic RNAs were isolated using the NE-PER nuclear and cytoplasmic extraction reagents (Thermo Fisher Scientific) based on the manufacturer’s instructions. For RNase R treatment, total RNA from BC cells was digested with 4 U/μg RNase R (Geneseed) at 70 °C for 10 min. RNA was reversely transcribed with the M-MLV Reverse Transcriptase (Promega, Madison, WI, USA) or miScripIIRT kit (Qiagen). Quantitative-PCR was executed with the SYBR Green PCR Master Mix (Vazyme, Nanjing, China). The data were analyzed by the 2^−ΔΔCt^ method. The primers utilized in the research were as follows: β-actin (Forward: 5′-CTTCGCGGGCGACGAT-3′); Reverse: 5′-CCACATAGGAATCCTTCTGACC-3′), circ_IRAK3: (Forward: 5′-CTCGGTCATCTGTGGCAGTA-3′; Reverse: 5′-GTGCCCAGGACCAAAGTAAT-3′), IRAK3: (Forward: 5′-AGGATTTCCGCGGTTGTGT-3′; Reverse: 5′-ACTCAACACTGCTCCCAGG-3′), KIF2A: (Forward: 5′-TCGTACCTGCATGATTGCCA-3′; Reverse: 5′-CCACTACCCTGTGAGAAGGG-3′, miR-603: (Forward: 5′-CGCGCACACACTGCAATTAC-3′; Reverse: 5′-AGTGCAGGGTCCGAGGTATT-3′, and U6 small nuclear RNA (U6): Forward: 5′-CTCGCTTCGGCAGCACA-3′; Reverse: 5′-AACGCTTCACGAATTTGCGT-3′. β-actin or U6 was used as an internal control.

### Cell cycle progression analysis

Flow cytometry assay was employed to assess cell cycle progression. In short, the transfected BC cells were harvested and fixed with cold ethanol (70%, Sigma) at 4 °C overnight. After washing with phosphate buffer saline (PBS, Thermo Fisher Scientific), the cells were treated with RNase A (100 μg/mL, Sigma) and stained with propidium iodide (PI) (50 μg/mL, Sigma). The cell distribution was evaluated with a flow cytometer (BD Biosciences, San Jose, CA, USA).

### Colony formation assay

BC cells (1 × 10^3^) were placed into 12-well plates and cultured for 2 weeks after specific vectors or oligonucleotides transfection. Thereafter, the cells were washed with PBS (Thermo Fisher Scientific) and stained with crystal violet (0.1%, ChemeGen, Shanghai, China). Finally, the colonies were photographed and counted with an inverted microscope (MTX Lab Systems, Bradenton, FL, USA).

### Cell proliferation analysis

BC cells (1 × 10^3^) were transfected with specific vectors or oligonucleotides and then seeded into 96-well plates. After culturing for 1 day, 2 days, or 3 days, the cells were incubated with 3-(4,5-dimethylthiazol-2-yl)-2,5-diphenyltetrazoliumbromide (MTT) solution (100 μL, 0.5 mg/mL, Sigma) for 4 h. Subsequently, the crystals were dissolved by dimethylsulfoxide (DMSO) (150 μL, Sigma). The absorbance at 570 nm was analyzed using the Microplate Reader (Bio-Rad, Hercules, CA, USA).

### Migration and invasion analysis

The transwell chambers (8 μm, Costar, Cambridge, MA, USA) were employed to analyze cell migration and invasion. It should be noted that transwell chambers used for invasion assay were precoated with Matrigel (Sigma). In brief, the upper chamber was supplemented with the serum-free medium containing transfected BC cells (1 × 10^5^ cells) and the bottom chamber was supplemented with a complete medium containing 10% FBS. 24 h later, the cells on the upper surface of the membrane were removed and the remaining cells were fixed with paraformaldehyde (4%, Solarbio) and stained with crystal violet (0.25%, ChemeGen). The average number of migrated and invaded cells in 5 random fields was counted, and these random fields were photographed with an inverted microscope (MTX Lab Systems) at 100 × magnification.

### Cell apoptosis analysis

The apoptosis of transfected BC cells was analyzed by flow cytometry assay with the Annexin V-Fluorescein Isothiocyanate (FITC)/PI Apoptosis Detection kit (Solarbio). In short, transfected BC cells were collected by centrifugation (1000×*g*, 5 min) and then re-suspended in binding buffer. Next, the cells were stained with Annexin V-FITC and PI for 30 min in the dark. Subsequently, the apoptotic rate was assessed with the flow cytometer (BD Biosciences).

### Western blotting

Total protein (tissue samples and cells) was extracted with the RIPA buffer containing a protease inhibitor cocktail (Roche, Basel, Switzerland). Sodium dodecyl sulfate–polyacrylamide gel electrophoresis (10%, SDS-PAGE, Sangon Biotech, Shanghai, China) was conducted to isolate total protein. Next, the isolated proteins were transferred to a polyvinylidene difluoride (PVDF) membrane (Roche) and then blocked with tris buffered saline tween (TBST) buffer containing 5% skim milk. After washing with TBST, the membranes were incubated with primary antibodies at 4 °C overnight, including anti-Cleaved PARP (#9541, 1:1000), anti-B-cell lymphoma/leukaemia-2 (Bcl-2) (#4223, 1:1000), anti-Bcl-2-associated x (Bax) (#2772, 1:1000), anti-KIF2A (ab197988, 1:200, Abcam, Cambridge, MA, USA), and anti-β-actin (#4967, 1:1000). Subsequently, the membranes were incubated with goat anti-rabbit IgG (#7077, 1:2000). Protein bands were visualized with an ImmunoStar LD (Wako Pure Chemical, Osaka, Japan). β-actin was used as a loading control. All antibodies were purchased from Cell Signaling Technology (Santa Cruz, California, USA), except for KIF2A.

### Bioinformatics analysis

The binding sites between circ_IRAK3 and miR-603 were predicted with the circBank (http://www.circbank.cn/) database. The binding sites of KIF2A in miR-603 were predicted using the miRDB database (http://mirdb.org/).

### Dual-luciferase reporter assay

The luciferase vectors carrying circ_IRAK3-wild type (WT), circ_IRAK3-mutant (MUT), KIF2A-WT, or KIF2A-MUT were established by the pMIR-REPORT™ reporter vector supplied by Genechem Co., Ltd. (Shanghai, China). BC cells (at logarithmic phase) were co-transfected with luciferase a reporter vector and miR-603 or miR-NC, followed by analyzing the luciferase activity using the luciferase reporter assay kit (Promega).

### RNA pull-down assay

Biotinylated circ_IRAK3-WT, circ_IRAK3-MUT, KIF2A-WT, KIF2A-MUT (bio-circ_IRAK3-WT, bio-circ_IRAK3-MUT, bio-KIF2A-WT, and bio-KIF2A-MUT) and scrambled negative control probe were synthesized by RiboBio (Guangzhou, China). For RNA pull-down assay, these probes were incubated with streptavidin magnetic beads (Thermo Fisher Scientific) to generate probe-coated beads. Afterward, these probe-coated beads were respectively incubated with the supernatant of the lysate of BC cells at 4 °C overnight. The pulled-down miRs were extracted using Trizol (Thermo Fisher Scientific) and then subjected to qRT-PCR.

### Xenograft assay

The protocols of xenograft assay were approved by the Animal Care Ethics Committee of the First Affiliated Hospital of Zhengzhou University. Tumor formation experiments were executed with 10 4-week-old female BALB/c nude mice (Vital River Laboratory, Beijing, China), which were randomly divided into 2 groups (n  = 5). The mice in each group were injected with MDA-MB-231 cells (about 5.0 × 10^6^) carrying sh-circ_IRAK3 or sh-NC. These mice were fed under Specific Pathogen Free conditions and tumor volume was measured every 5 days with a caliper from day 10 [Volume = (length × width^2^)/2]. On the 35th day of injection, all mice were anesthetized with xylazine (10 mg/kg, ChemeGen) and then euthanized by cervical decapitation. The tumor tissue was excised for subsequent analysis after the mice died (pupil dilation and cardiac arrest).

### Statistical analysis

All data analyses were conducted with the SPSS 20.0 software (SPSS, Chicago, IL, USA). Data were shown as the mean ± standard deviation. The correlation among circ_IRAK3, miR-603, or KIF2A was assessed by Pearson’s correlation analysis. The experiments in vitro were repeated at least 3 times. The differences between the two groups were determined with paired Student’s *t *test or independent Student’s *t* test. One-way analysis of variance with Tukey’s post hoc test was employed to assess the differences among 3 or more groups. *P*  < 0.05 was defined as a significant difference.

## Results

### Circ_IRAK3 was highly expressed in BC tissues and cells

**Fig. 1:**
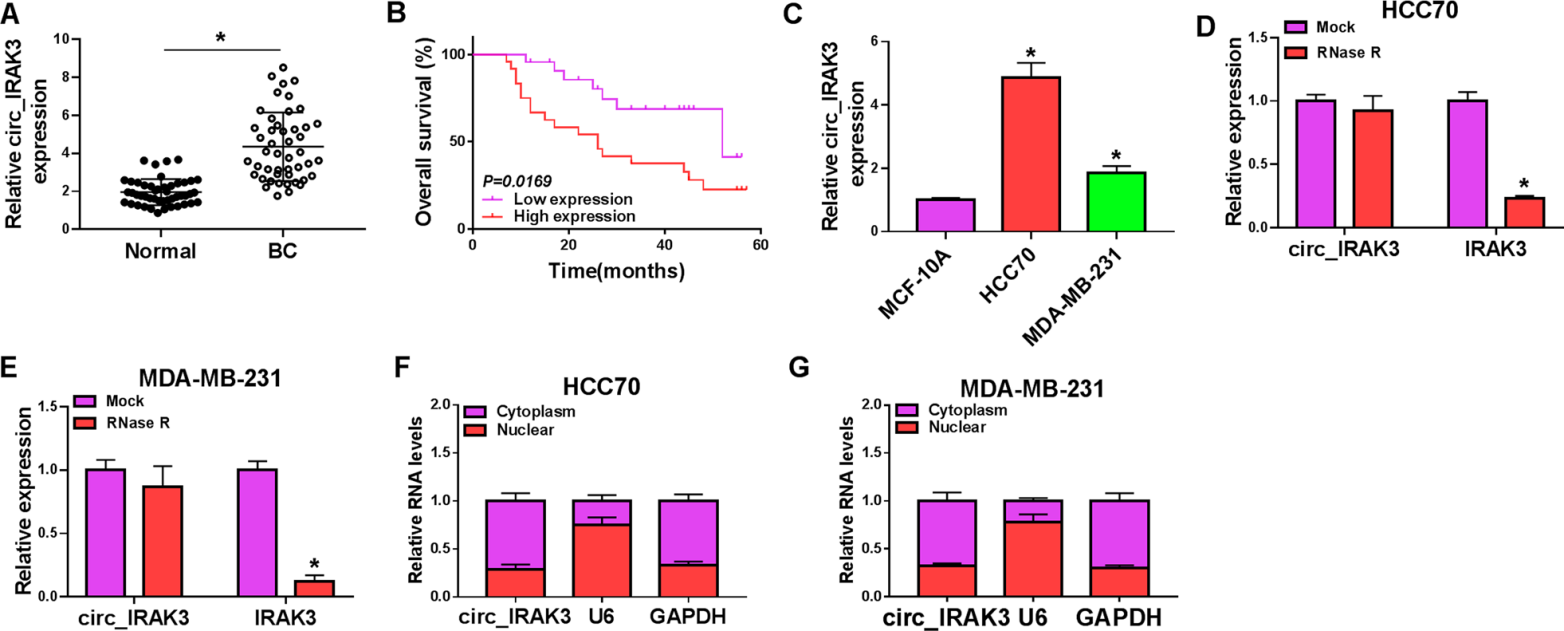
Expression pattern of circ_IRAK3 in BC tissues and cells. **A** The expression of circ_IRAK3 in BC tissues (compared to adjacent non-cancerous tissues) was estimated by qRT-PCR. **B** Kaplan–Meier curves of overall survival for BC patients with high or low expression of circ_IRAK3. The cutoff value was the median of circ_IRAK3 expression in 47 BC patients, of which 23 were classified as low expression and 24 were classified as high expression. **C** Relative expression of circ_IRAK3 in BC cells (HCC70 and MDA-MB-231) (compared with MCF-10A cells) was evaluated by qRT-PCR. **D**, **E** The levels of circ_IRAK3 and IRAK3 mRNA in total RNA of HCC70 and MDA-MB-231 under RNase R treatment were analyzed by qRT-PCR. **F**, **G** Following the nuclear-cytoplasmic fractionation assay, the abundance of circ_IRAK3 in the nuclear and cytoplasmic portions was analyzed by qRT-PCR. *P  < 0.05

To confirm the differential expression of circ_IRAK3 in BC, we detected the level of circ_IRAK3 in 47 pairs of BC tissues and adjacent non-cancerous tissues by qRT-PCR. In comparison to the adjacent non-cancerous tissues, circ_IRAK3 expression was observably higher in BC tissues (Fig. 1A). High expression of circ_IRAK3 was associated with TNM grade, lymph node metastasis, and tumor size of BC patients (Table 1). Also, BC patients with high expression of circ_IRAK3 had a lower overall survival (Fig. 1B). Circ_IRAK3 was generated by the back-splicing of exons 2–7 of the IRAK3 gene, which was confirmed by sequencing (Additional file 2: Fig. S2A, B). Furthermore, circ_IRAK3 expression was markedly elevated in BC cells (HCC70 and MDA-MB-231) in contrast to the MCF-10A cells (Fig. 1C). Subsequently, we further verified the circular characteristics of circ_IRAK3 in HCC70 and MDA-MB-231 cells through RNase R treatment. As displayed in Fig. 1D, E, linear IRAK3 mRNA was observably digested by RNase R treatment, while circ_IRAK3 was resistant to RNase R. Nuclear-cytoplasmic fractionation assays showed that circIRAK3 was more localized in the cytoplasm of BC cells (Fig. 1F, G). Collectively, circ_IRAK3 was overexpressed in BC.

**Table 1:** Relationship between circ_IRAK3 expression and clinicopathologic features of breast cancer patients

	Characteristics n = 47	circ_IRAK3 expression		*P* value^a^
		Low (n = 23)	High (n = 24)	
Age (years)				0.2476
≤ 50	22	13	9	
> 50	25	10	15	
TNM grade				0.0032*
I + II	20	15	5	
III	27	8	19	
Lymph node metastasis				0.0087*
Positive	24	7	17	
Negative	23	16	7	
Tumor size				0.0012*
≤ 2 cm	23	17	6	
> 2 cm	24	6	18	
ER status				0.5639
Positive	22	12	10	
Negative	25	11	14	
PR status				0.7725
Positive	25	13	12	
Negative	22	10	12	
HER-2 status				0.2476
Positive	23	9	14	
Negative	24	14	10	

*TNM* tumor-node-metastasis

^a^Chi-square test

*P  < 0.05

### Silencing of circ_IRAK3 induced apoptosis, impeded proliferation, migration, and invasion of BC cells

**Fig. 2:**
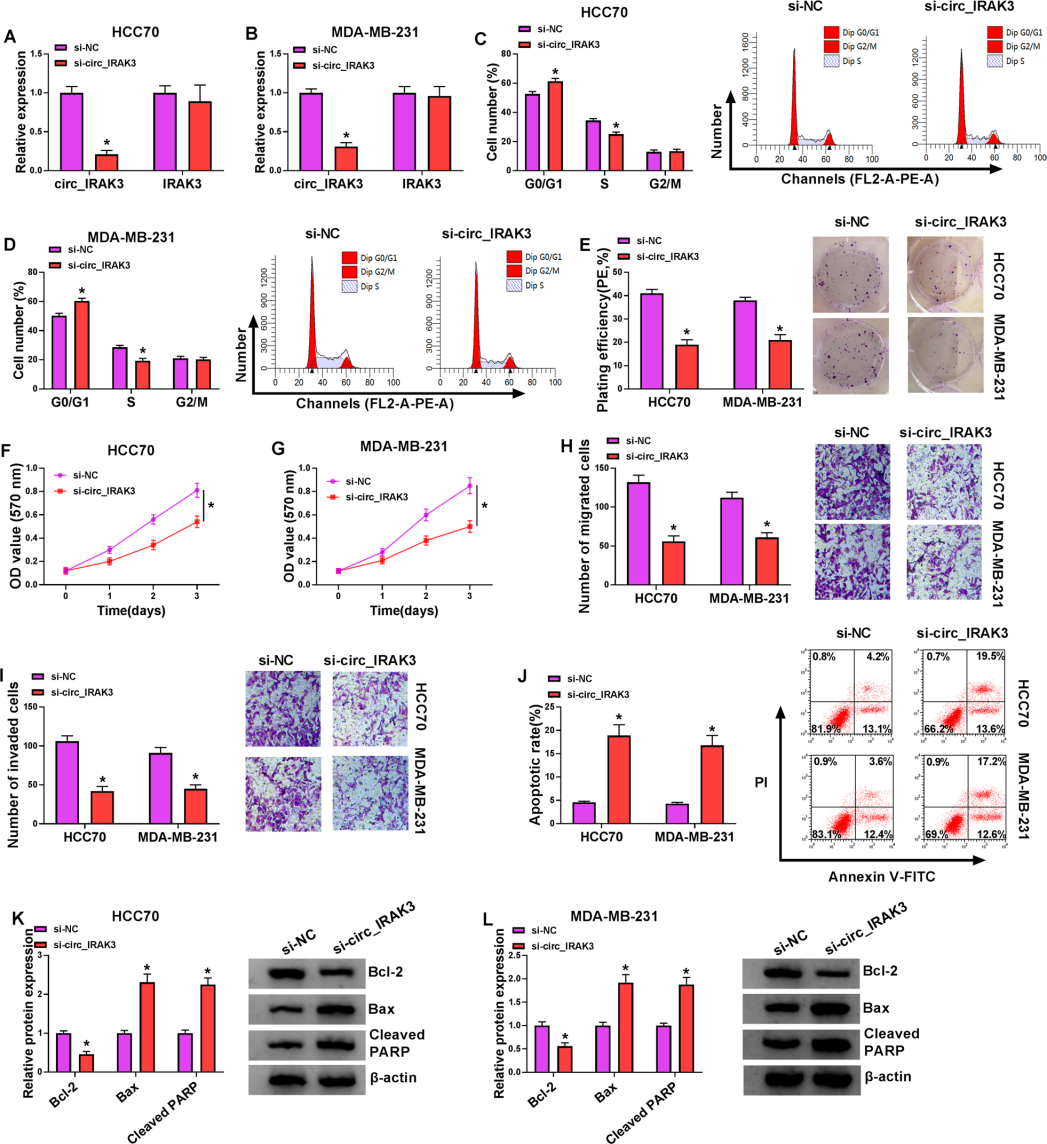
Impacts of circ_IRAK3 inhibition on malignant behaviors of BC cells. **A**–**L** HCC70 and MDA-MB-231 cells were transfected with si-NC or si-circ_IRAK3. **A**, **B** QRT-PCR was employed to assess the levels of circ_IRAK3 and IRAK3 mRNA in HCC70 and MDA-MB-231 cells. **C**–**G** The cell cycle progression, colony formation, and proliferation were analyzed by flow cytometry, colony formation, or MTT assays. **H**–**J** The migration, invasion, and apoptosis of HCC70 and MDA-MB-231 cells were assessed by transwell or flow cytometry assays. **K**, **L** The levels of Bax, Cleaved PARP, and Bcl-2 in HCC70 and MDA-MB-231 cells were examined by western blotting. *P  < 0.05

To survey the biological role of circ_IRAK3 in BC cells, we silenced the expression of circ_IRAK3 through transfection of si-NC or si-circ_IRAK3 into BC cells. The data exhibited that circ_IRAK3 expression was apparently reduced in HCC70 and MDA-MB-231 cells after si-circ_IRAK3 transfection, while IRAK3 mRNA expression did not change (Fig. 2A, B). Then, we explored the impacts of circ_IRAK3 inhibition on the malignant behaviors of BC cells. Cell cycle analysis presented that circ_IRAK3 knockdown elevated the percentage of HCC70 and MDA-MB-231 cells in the G0/G1 stage and reduced the percentage of HCC70 and MDA-MB-231 cells in the S stage, manifesting that circ_IRAK3 silencing could arrest cell cycle progression in HCC70 and MDA-MB-231 cells (Fig. 2C, D). Colony formation assay showed that silenced circ_IRAK3 expression decreased the colony formation ability of HCC70 and MDA-MB-231 cells (Fig. 2E). MTT assay presented that circ_IRAK3 knockdown curbed cell proliferation in HCC70 and MDA-MB-231 cells (Fig. 2F, G). Transwell assay exhibited that circ_IRAK3 silencing led to a decrease in the number of migrating and invading cells (Fig. 2H, I). Cell apoptosis analysis indicated that circ_IRAK3 downregulation elevated cell apoptotic rate in HCC70 and MDA-MB-231 cells (Fig. 2J). We also detected the levels of apoptosis-related markers Bax, Cleaved PARP, and Bcl-2. Also, western blotting revealed that the levels of Bax and Cleaved PARP were increased in circ_IRAK3-silenced HCC70 and MDA-MB-231 cells, while the level of Bcl-2 was decreased (Fig. 2K, L). These results manifested that circ_IRAK3 knockdown suppressed the malignant behaviors of BC cells.

### Circ_IRAK3 was verified as a sponge for miR-603 in BC cells

**Fig. 3:**
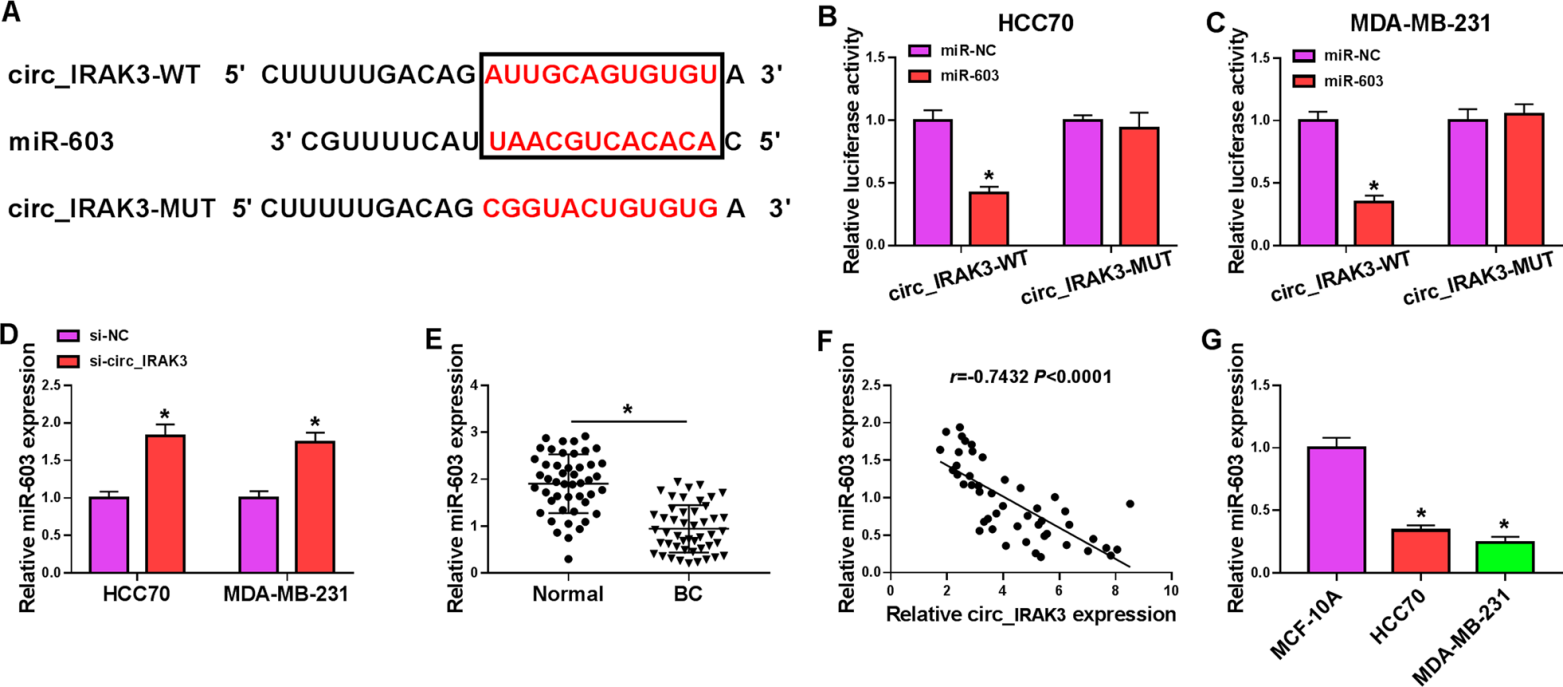
Circ_IRAK3 acted as a sponge for miR-603 in BC cells. **A** The schematic drawing exhibited the putative binding sites between miR-603 and circ_IRAK3. **B**, **C** Dual-luciferase reporter assay was conducted to verify the relationship between miR-603 and circ_IRAK3 in HCC70 and MDA-MB-231 cells. **D** QRT-PCR revealed the expression of miR-603 in HCC70 and MDA-MB-231 cells transfected with si-NC or si-circ_IRAK3. **E** QRT-PCR exhibited the expression of miR-603 in BC tissues and adjacent non-cancerous tissues. **F** Pearson’s correlation analysis presented the correlation between miR-603 and circ_IRAK3 in BC tissues. **G** QRT-PCR presented miR-603 expression in BC cells and the MCF-10A cells. *P  < 0.05

Researchers have demonstrated that circRNAs can function as “sponges” to miRs [23]. The circBank database predicted that there were multiple miRs that may interact with circIRAK3. 4 miRNAs (miR-515-5p [24], miR-135a-5p [25], miR-135b-5p [26], and miR-603 [19]) that were found to be low-expressed in BC through literature review were selected for further analysis. Among these 4 miRs, miR-603 was markedly upregulated in si-circ_IRAK3-transfected BC cells (Additional file 3: Fig. S3A, B). The putative binding sites between miR-603 and circ_IRAK3 were presented in Fig. 3A. Dual-luciferase reporter assay indicated that the luciferase activity in HCC70 and MDA-MB-231 cells co-transfected with luciferase vectors carrying circ_IRAK3-WT and miR-603 mimic was decreased, while there was no overt difference in HCC70 and MDA-MB-231 cells co-transfected with luciferase reporter vectors containing circ_IRAK3-MUT and miR-603 mimic (Fig. 3B, C). Also, miR-603 could be pulled down by bio-circ_IRAK3-WT probe instead of bio-NC and bio-circ_IRAK3-MUT probes (Additional file 4: Fig. S4A). Also, the abundance of circ_IRAK3 and miR-603 was higher in the anti-Ago2 group than that in the anti-IgG group (Additional file 5: Fig. S5A, B). As expected, suppression of circ_IRAK3 increased the expression of miR-603 in HCC70 and MDA-MB-231 cells compared to the control group (Fig. 3D). Moreover, miR-603 expression was overtly lower in BC tissues than adjacent non-cancerous tissues (Fig. 3E). Also, the expression of miR-603 had a negative correlation with circ_IRAK3 in BC tissues (Fig. 3F). Consistently, miR-603 was downregulated in BC cells than that in the MCF-10A cells (Fig. 3G). These findings indicated that circ_IRAK3 might function as a miR-603 sponge in BC cells.

### MiR-603 silencing overturned circ_IRAK3 knockdown-mediated effects on malignant behaviors of BC cells

**Fig. 4:**
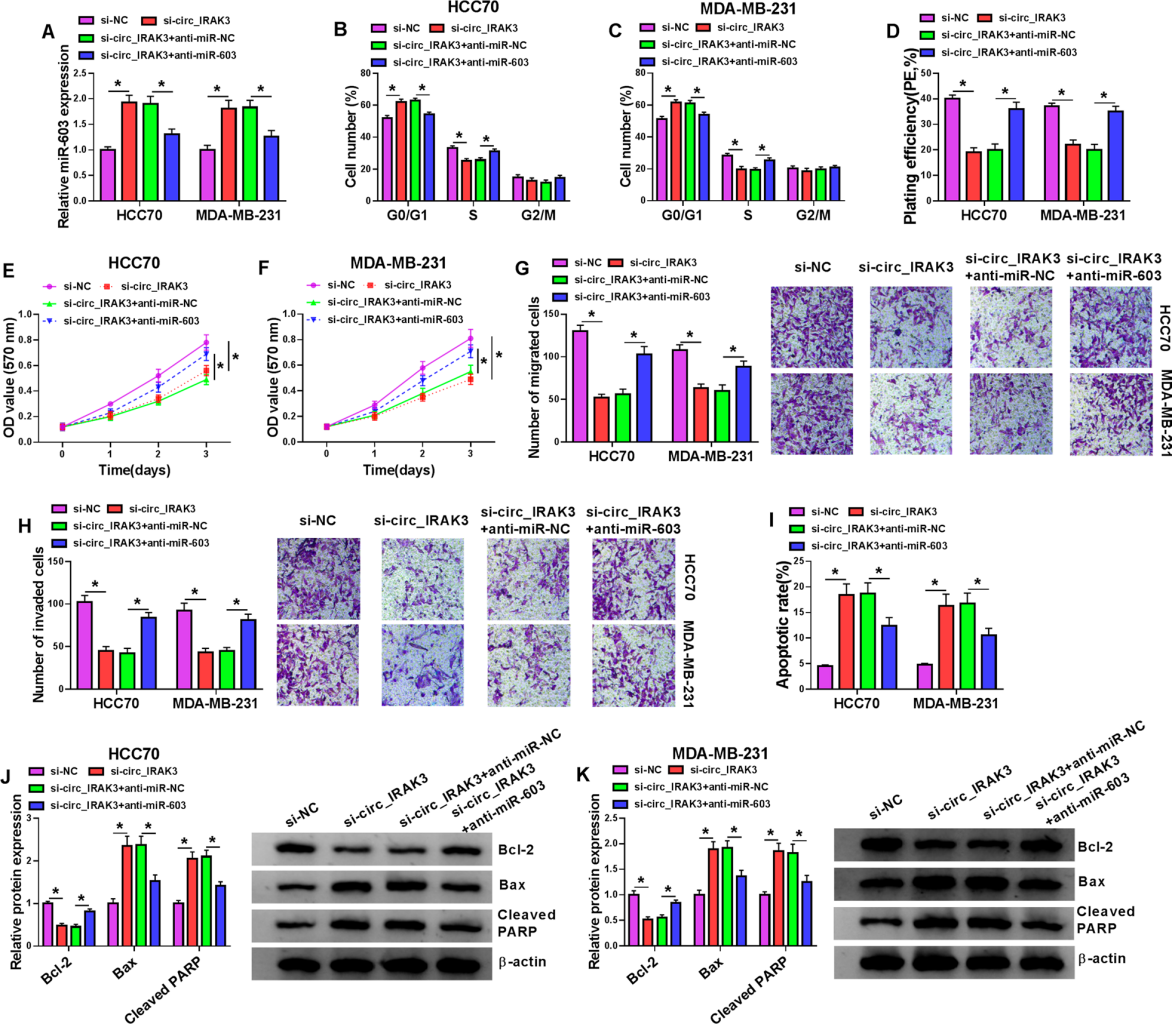
Circ_IRAK3 exerted a role in BC via miR-603. **A**–**K** HCC70 and MDA-MB-231 cells were transfected with si-NC, si-circ_IRAK3, si-circ_IRAK3 + anti-miR-NC, or si-circ_IRAK3 + anti-miR-603. **A** Expression of miR-603 in HCC70 and MDA-MB-231 cells was surveyed by qRT-PCR. **B**–**F** Flow cytometry, colony formation, and MTT assays were performed to evaluate the cell cycle progression, colony formation, or proliferation of HCC70 and MDA-MB-231 cells. **G**–**I** Transwell and flow cytometry assays were conducted to analyze cell migration, invasion, or apoptosis in HCC70 and MDA-MB-231 cells. **J**, **K** Western blotting revealed the levels of Bax, Cleaved PARP, and Bcl-2 in HCC70 and MDA-MB-231 cells. *P < 0.05

Based on the above findings, we further analyzed whether miR-603 was related to the malignant behaviors of BC cells mediated by circ_IRAK3. We observed that the upregulation of miR-603 in HCC70 and MDA-MB-231 cells caused by circ_IRAK3 inhibition was reversed after transfection with miR-603 inhibitor (Fig. 4A). Moreover, the suppressive impacts of circ_IRAK3 silencing on cell cycle progression, colony formation, proliferation, migration, and invasion of HCC70 and MDA-MB-231 cells were overturned by miR-603 inhibitor (Fig. 4B–H). As expected, circ_IRAK3 knockdown decreased N-cadherin and vimentin protein levels and increased E-cadherin protein levels, but these changes were weakened by anti-miR-603 introduction (Additional file 6: Fig. S6A, B). Also, silenced miR-603 expression impaired the elevation of the apoptotic rate of HCC70 and MDA-MB-231 cells induced by circ_IRAK3 silencing (Fig. 4I). Additionally, miR-603 silencing overturned the effects of circ_IRAK3 inhibition on Bax, Cleaved PARP, Bcl-2 protein levels in HCC70 and MDA-MB-231 cells (Fig. 4J, K). These findings suggested that circ_IRAK3 promoted BC cell malignant behaviors through adsorbing miR-603.

### KIF2A was a downstream target of miR-603

**Fig. 5:**
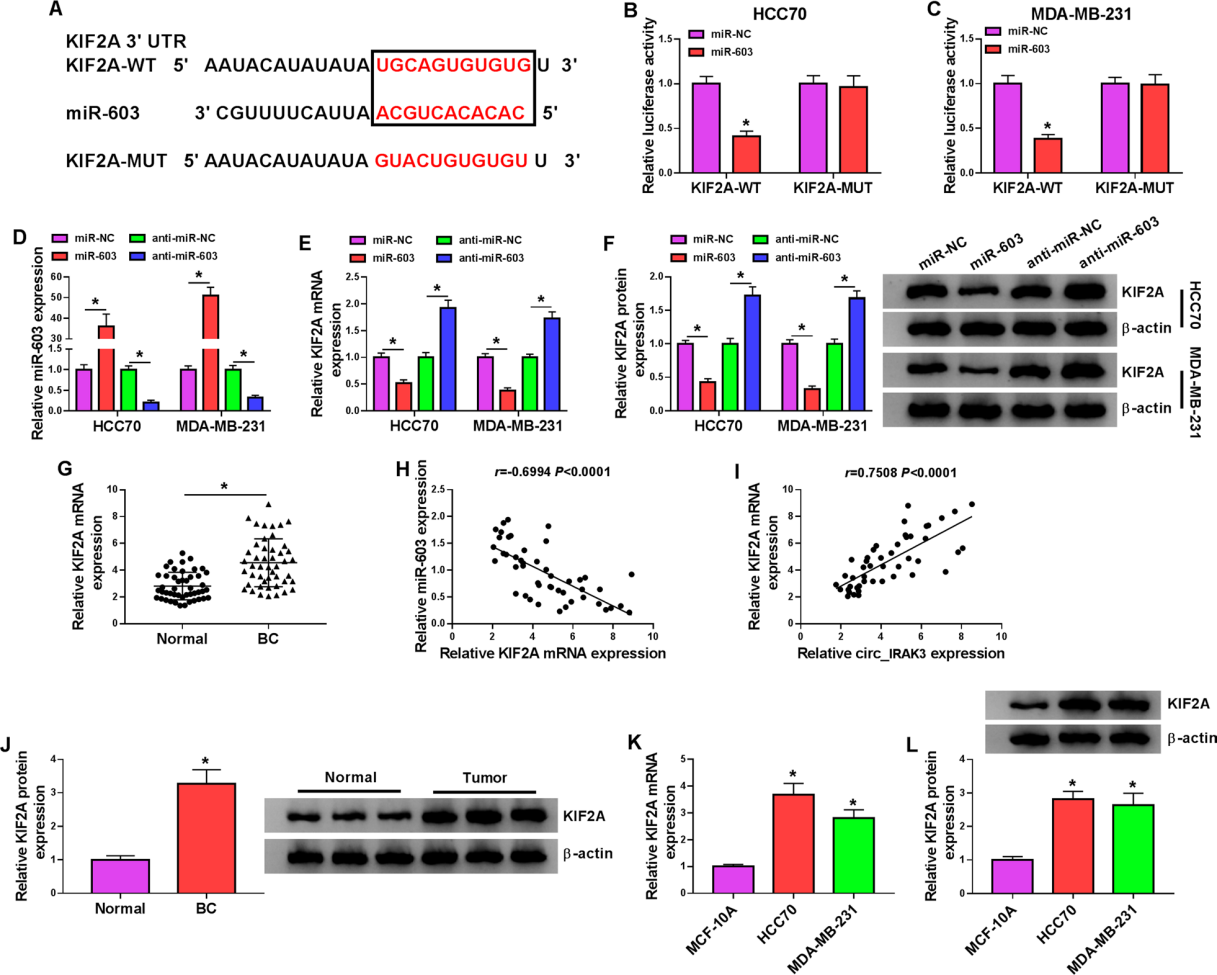
KIF2A acted as a downstream target of miR-603. **A** Schematic representation of the putative binding sites between KIF2A and miR-603. **B**, **C** Dual-luciferase reporter assay was employed to determine the relationship between KIF2A and miR-603. **D** QRT-PCR presented the transfection efficiency of miR-603 mimic and inhibitor. **E**, **F** Influence of miR-603 overexpression or silencing on the levels of KIF2A mRNA and protein in HCC70 and MDA-MB-231 cells was analyzed by qRT-PCR or western blotting. **G** The level of KIF2A mRNA in BC tissues and adjacent non-cancerous tissues was assessed with qRT-PCR. **H**, **I** The correlation between KIF2A and miR-603 or circ_IRAK3 was assessed with Pearson’s correlation analysis. **J** Western blotting was used to analyze the level of KIF2A protein in BC tissues and adjacent non-cancerous tissues. **K**, **L** QRT-PCR and western blotting were performed to evaluate the levels of KIF2A mRNA and protein in BC cells and the MCF-10A cells. *P < 0.05

Subsequently, we predicted the downstream targets of miR-603 using the miRDB database. 6 genes (NFIB [27], ZEB2 [28], TLR4 [29], KDM7A [30], LASP1 [31], and KIF2A [32]) related to BC were selected as candidate targets for further analysis among all predicted target genes. Moreover, only KIF2A was repressed in both miR-603-overexpressing HCC70 and MDA-MB-231 cells, so KIF2A was selected for subsequent investigation (Additional file 3: Fig. S3C, D). The putative binding sites between miR-603 and KIF2A were displayed in Fig. 5A. Moreover, the luciferase activity of luciferase vectors containing KIF2A 3′UTR-WT was repressed in HCC70 and MDA-MB-231 cells transfected with miR-603 mimic, but the luciferase activity of luciferase vectors with KIF2A 3′UTR-MUT was not affected by miR-603 mimic (Fig. 5B, C). As expected, miR-603 could be pulled down by bio-KIF2A-WT but not bio-NC and bio-KIF2A-MUT probes (Additional file 4: Fig. S4B). There were higher levels of miR-603 and KIF2A mRNA in the anti-Ago2 group than those in the anti-IgG group (Additional file 5: Fig. S5C, D). We also observed that miR-603 expression was elevated in miR-603 mimic-transfected HCC70 and MDA-MB-231 cells and reduced in miR-603 inhibitor-transfected HCC70 and MDA-MB-231 cells (Fig. 5D). Furthermore, the mRNA and protein levels of KIF2A in HCC70 and MDA-MB-231 cells were elevated by miR-603 inhibition and reduced by miR-603 overexpression (Fig. 5E, F). However, the upregulation of KIF2A had no effect on the expression of miR-603 (Additional file 7). Compared to the adjacent non-cancerous tissues, the level of KIF2A mRNA was overtly upregulated in BC tissues (Fig. 5G). The expression of KIF2A mRNA in BC tissues was negatively correlated with miR-603 and positively correlated with circ_IRAK3 (Fig. 5H, I). Also, the level of KIF2A protein was elevated in BC tissues (Fig. 5J). As expected, the mRNA and protein levels of KIF2A were also upregulated in BC cells when compared with the MCF-10A cells (Fig. 5K, L). Taken together, these data indicated that KIF2A might act as a target for miR-603 in BC cells.

### KIF2A overexpression reversed miR-603 mimic-mediated impacts on malignant behaviors of BC cells

**Fig. 6:**
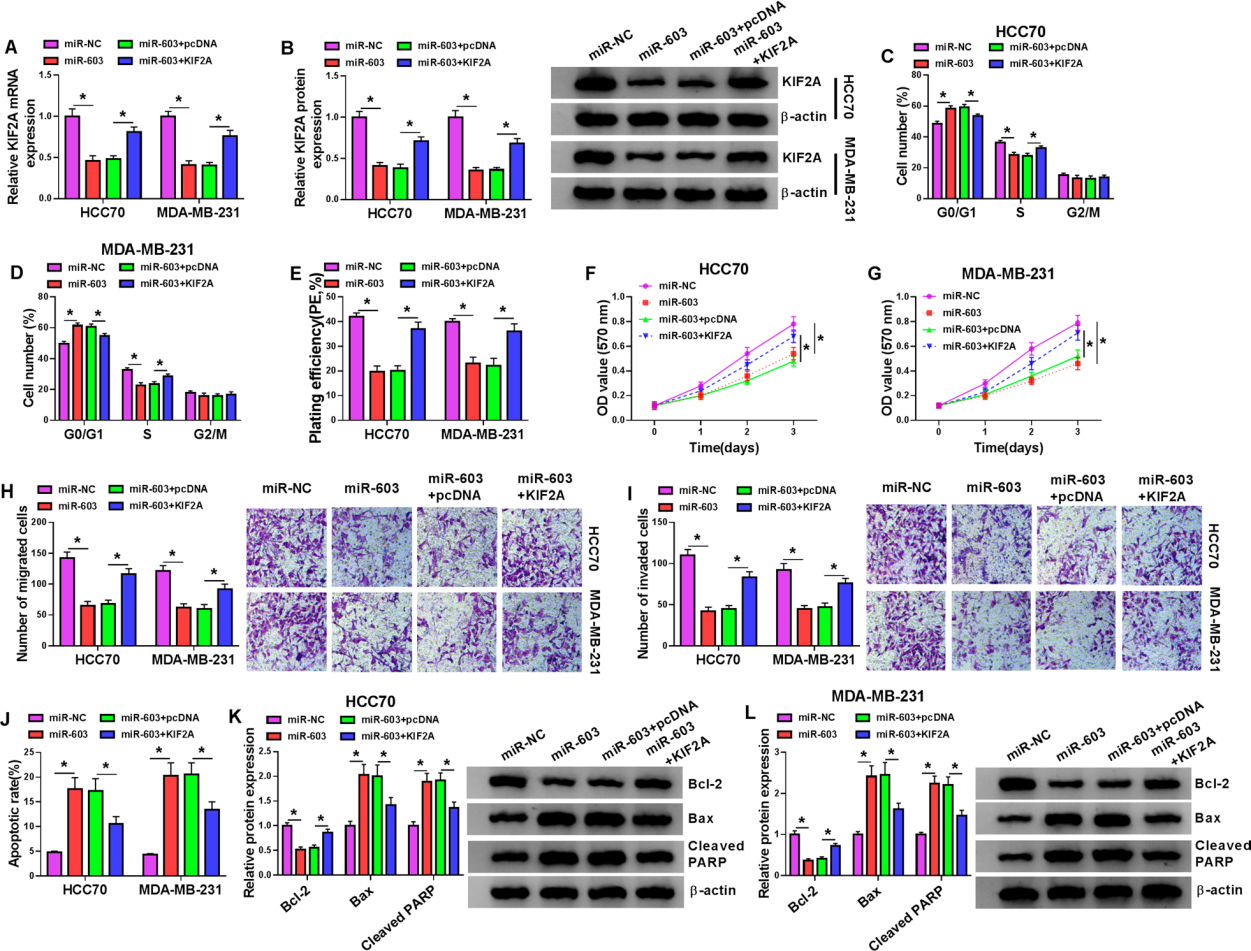
MiR-603 played a role in BC by targeting KIF2A. **A**–**L** HCC70 and MDA-MB-231 cells were transfected with miR-NC, miR-603, miR-603 + pcDNA, or miR-603 + KIF2A. **A**, **B** The levels of KIF2A mRNA and protein in HCC70 and MDA-MB-231 cells were detected with qRT-PCR or western blotting. **C**–**G** The cell cycle progression, colony formation, and proliferation of HCC70 and MDA-MB-231 cells were examined with flow cytometry, colony formation, or MTT assays. **H**–**J** The migration, invasion, and apoptosis of HCC70 and MDA-MB-231 cells were determined with transwell or flow cytometry assays. **K**, **L** Western blotting was conducted to assess the levels of Bax, Cleaved PARP, and Bcl-2 in HCC70 and MDA-MB-231 cells. *P  < 0.05

To check on whether miR-603 regulated malignant behaviors of BC cells via targeting KIF2A, HCC70 and MDA-MB-231 cells were transfected with miR-NC, miR-603, miR-603 + pcDNA, or miR-603 + KIF2A. The results exhibited that miR-603 overexpression repressed the levels of KIF2A mRNA and protein in HCC70 and MDA-MB-231 cells, but this influence was reversed by forcing KIF2A expression (Fig. 6A, B). We also observed that elevated KIF2A expression abolished the inhibiting impacts of miR-603 mimic on cell cycle progression, colony formation, proliferation, migration, and invasion of HCC70 and MDA-MB-231 cells (Fig. 6C–I). Furthermore, miR-603 overexpression reduced N-cadherin and vimentin protein levels and elevated E-cadherin protein levels, but these effects caused by miR-603 upregulation were impaired after KIF2A introduction (Additional file 5: Fig. S5C, D). Moreover, the promoting influence of miR-603 overexpression on the apoptosis of HCC70 and MDA-MB-231 cells was overturned after KIF2A introduction (Fig. 6J). Also, miR-603 overexpression elevated the levels of Bax and Cleaved PARP and reduced the level of Bcl-2 in HCC70 and MDA-MB-231 cells, but these tendencies were restored by KIF2A elevation (Fig. 6K, L). Collectively, these data indicated that miR-603 modulated malignant behaviors of BC cells via targeting KIF2A.

### Circ_IRAK3 modulated KIF2A expression through miR-603 in BC cells

**Fig. 7:**
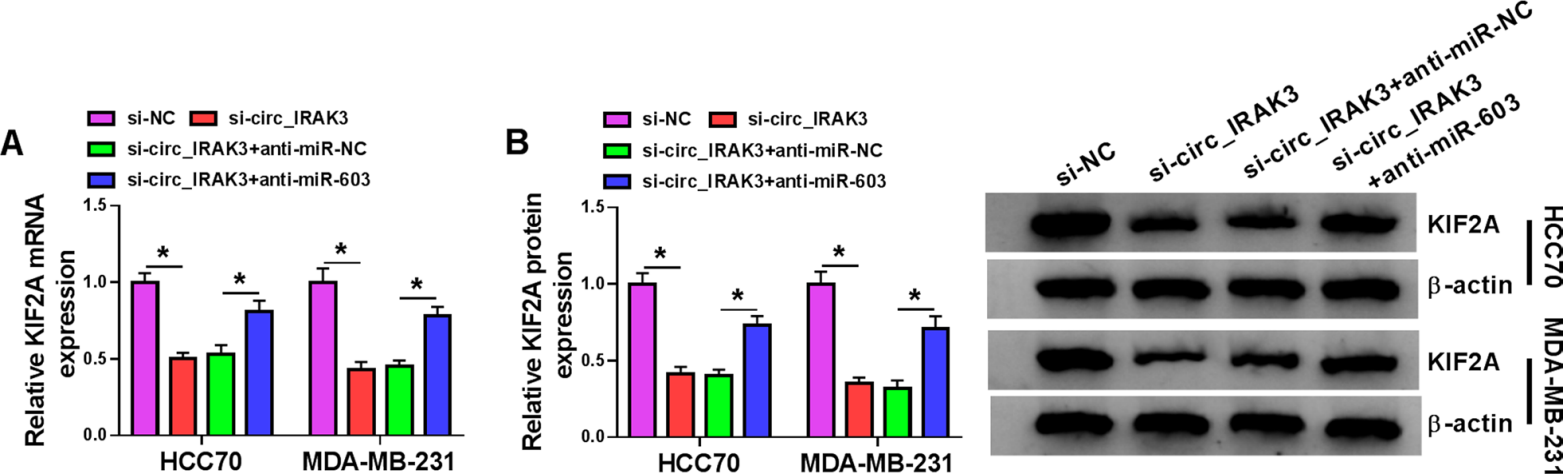
KIF2A expression was regulated by the circ_IRAK3/miR-603 axis. **A**, **B** QRT-PCR and western blotting displayed the levels of KIF2A mRNA and protein in HCC70 and MDA-MB-231 cells transfected with si-NC, si-circ_IRAK3, si-circ_IRAK3 + anti-miR-NC, or si-circ_IRAK3 + anti-miR-603. *P  < 0.05

In view of the targeting relationship between circ_IRAK3 or KIF2A and miR-603 in BC cells, we further verified whether circ_IRAK3 regulated KIF2A expression via sponging miR-603. The results exhibited that circ_IRAK3 silencing downregulated the levels of KIF2A mRNA and protein in HCC70 and MDA-MB-231 cells, but this trend was abolished by silence of miR-603 expression (Fig. 7A, B). These results indicated that circ_IRAK3 might modulate KIF2A expression through sponging miR-603.

### Silence of circ_IRAK3 decreased BC growth in vivo

**Fig. 8:**
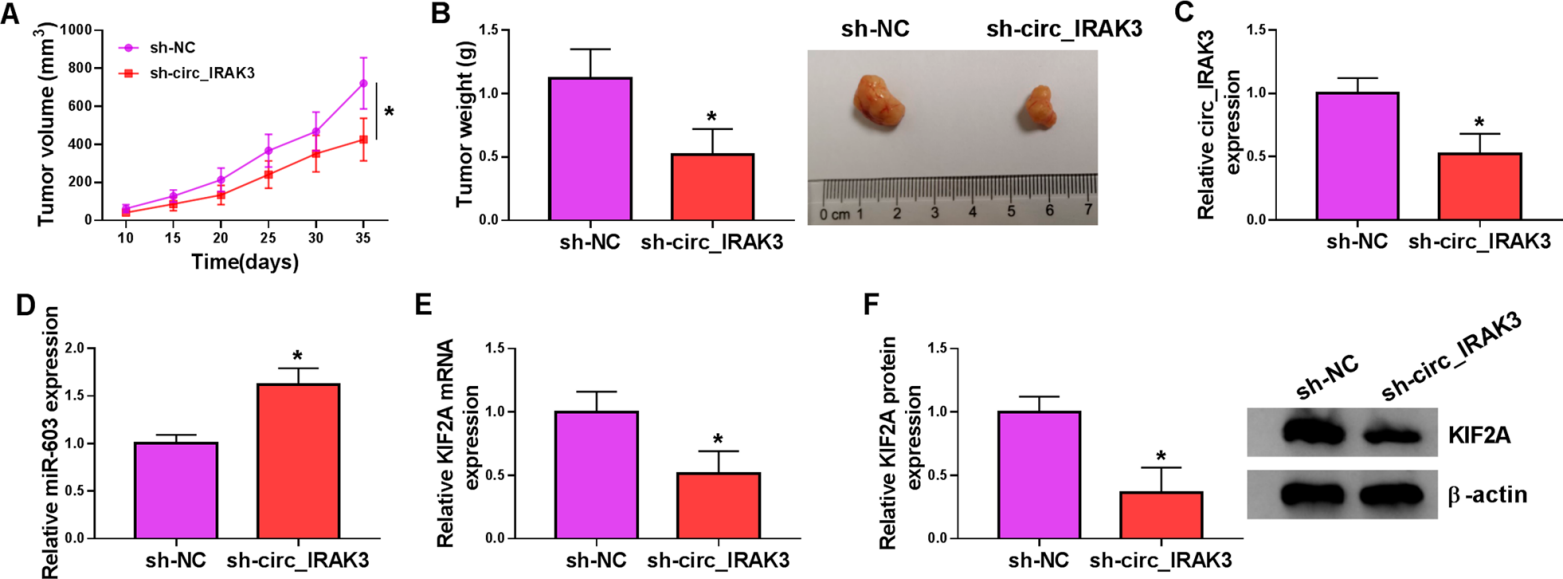
Circ_IRAK3 silencing reduced BC growth in vivo. **A** Tumor volume was monitored every 5 days from day 10. **B** Mice tumor tissues were photographed and weighted after 35 days of injection. **C**, **D** The expression of circ_IRAK3 and miR-603 in mice tumor tissues was measured by qRT-PCR. **E**, **F** The levels of KIF2A mRNA and protein in mice tumor tissues were assessed by qRT-PCR or western blotting. *P  < 0.05

To confirm the role of circ_IRAK3 in BC in vivo, we injected MDA-MB-231 cells that stably knockdown circ_IRAK3 into mice, and sh-NC was used as a control. The results exhibited that tumor volume and weight were restrained in the sh-circ_IRAK3 group in contrast to the sh-NC group (Fig. 8A, B, and Additional file 8: Fig. S8). Moreover, the expression of circ_IRAK3 was markedly reduced in mice tumor tissues of the sh-circ_IRAK3 group compared to the control group, while the expression of miR-603 was overtly increased (Fig. 8C, D). We also observed that the levels of KIF2A mRNA and protein were apparently decreased in mice tumor tissues of the sh-circ_IRAK3 group (Fig. 8E, F). These data suggested that circ_IRAK3 silencing could restrain BC growth in vivo.

## Discussion

BC is a malignant tumor that threatens the health of women worldwide [33, 34]. CircRNAs are promising tumor therapeutic targets and diagnostic biomarkers due to their unique structure [35]. A previous study indicated that circ_IRAK3 was upregulated in hepatocellular carcinoma [12]. Moreover, circ_IRAK3 was apparently higher in BC tissues, and circ_IRAK3 silencing reduced lung metastasis in vivo and repressed MDA-MB-231-LM2 cell migration and invasion via decreasing FOXC1 expression by sponging miR-3607 [13]. In line with a previous study, we also verified that circ_IRAK3 expression was increased in BC tissues and cells. Also, circ_IRAK3 inhibition reduced tumor growth in vivo and induced cell cycle arrest, apoptosis, impeded colony formation, proliferation, migration, and invasion of BC cells in vitro. These data indicated that circ_IRAK3 acted as an unfavorable circRNA in BC. However, Wu et al. suggested that circ_IRAK3 silencing had no effect on MDA-MB-231-LM2 cell proliferation, colony formation, and cell cycle progression [13]. Our results were partially different from Wu et al.’s research, which might be related to the difference in cell lines. Based on this difference, the biological function of circ_IRAK3 in BC needs to be further explored in the future.

Accumulated evidence had proved that circRNAs could sponge miRs to participate in the progression of several tumors. For example, circRNA hsa_circ_0000670 facilitated gastric cancer development through regulating SIX4 expression via sponging miR-384 [36]. Also, circ_IRAK3 could sponge miR-3607 to accelerate BC progression [13]. Herein, we discovered that circ_IRAK3 served as a sponge for miR-603 in BC cells. MiR-603 had been revealed to play different roles in different tumors. One report uncovered that miR-603 repressed malignant behaviors of ovarian cancer cells by targeting HK-2 [37]. Bayraktar et al. discovered that miR-603 played a tumor-suppressive role in BC through the regulation of eEF2K expression [19]. However, miR-603 was discovered to play a cancerigenic role in glioma [38] and osteosarcoma [39], and hepatocellular carcinoma [40]. In the research, we verified that miR-603 acted as a tumor repressor in BC, which was in line with the study of Bayraktar et al. [19]. Also, miR-603 silencing overturned the inhibitory impact of circ_IRAK3 downregulation on cell malignant behaviors in BC cells. Therefore, we deduced that circ_IRAK3 regulated BC development through adsorbing miR-603.

**Fig. 9:**
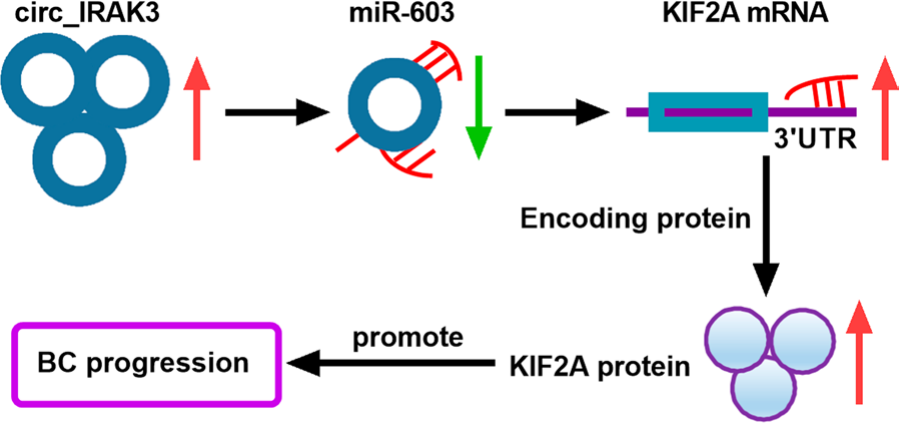
A schematic model showing the mechanism involved in BC cell malignant behaviors mediated by circ_IRAK3. Circ_IRAK3 adsorbed miR-603, thereby releasing the inhibitory effect of miR-603 on its target gene KIF2A and increasing the levels of KIF2A mRNA and protein, leading to the promotion of BC progression

KIF2A exerts a key role in the regulation of microtubule dynamics in the process of mitosis [41, 42]. Researchers had proved that KIF2A served as an oncogene in a series of tumors, such as nasopharyngeal carcinoma [22], lung adenocarcinoma [43], gastric cancer [44], and glioma [45]. Wang et al. disclosed that KIF2A was associated with poor prognosis of BC patients, and KIF2A silencing curbed migration and proliferation of BC cells [32]. Herein, KIF2A was verified as a target of miR-603 in BC cells. Moreover, KIF2A overexpression overturned the repressive impact of miR-603 mimic on the malignancy of BC cells. Also, circ_IRAK3 could modulate KIF2A expression by sponging miR-603 in BC cells. Therefore, we concluded that circ_IRAK3 modulated BC development through modulating KIF2A expression via sponging miR-603 (Fig. 9).

## Conclusion

Circ_IRAK3 was overexpressed in BC. Moreover, circ_IRAK3 knockdown induced cell proliferation, and repressed cell proliferation, metastasis, and invasion in BC cells. Importantly, circ_IRAK3 could adsorb miR-603 to regulate KIF2A expression. Thus, we inferred that circ_IRAK3 facilitated BC cell malignant behaviors through KIF2A by sponging miR-603. The research offered evidence to support the cancerogenic role of circ_IRAK3 in BC, manifesting that circ_IRAK3 was a possible target for BC treatment. The limitation of this study is that the downstream pathway of the circ_IRAK3/miR-603/KIF2A axis has not been investigated, which can be investigated in the future. In addition, the small number and weak representativeness of clinical samples are the difficulties of this study, so the role of circ_IRAK3 in BC needs to be further confirmed.

## Supplementary Information

**Additional file 1: Figure S1.** The experimental flowchart of this study.

**Additional file 2: Figure S2.** **A** A schematic diagram of circ_IRAK3 generated by the back-splicing of exons 2–7 of the IRAK3 gene. **B** Schematic diagram exhibiting a partial figure of the circ_IRAK3 sequencing result (the back-splicing region of circ_IRAK3).

**Additional file 3: Figure S3.** **A**, **B** QRT-PCR was performed to analyze the effect of Circ_IRAK3 silencing on the relative levels of 4 miRNAs (miR-515-5p, miR-135a-5p, miR-135b-5p, and miR-603). **C**, **D** QRT-PCR analysis of the effect of miR-603 overexpression on the relative levels of 6 candidate targets (NFIB, ZEB2, TLR4, KDM7A, LASP1, and KIF2A). *P < 0.05.

**Additional file 4: Figure S4.** RNA pull-down assay analysis of the relationship between circ_IRAK3 or KIF2A and miR-603. *P < 0.05.

**Additional file 5: Figure S5.** RIP assays showing the relationship circ_IRAK3 or KIF2A and miR-603. *P < 0.05.

**Additional file 6: Figure S6.** Western blotting exhibiting E-cadherin, N-cadherin, and vimentin protein levels in transfected HCC70 and MDA-MB-231 cells. *P < 0.05.

**Additional file 7: Figure S7.** Effect of KIF2A overexpression on the expression of miR-603 in BC cells was determined by qRT-PCR.

**Additional file 8: Figure S8.** A picture showing other xenograft tumors not shown in Fig. 8.

## Data Availability

All data generated or analyzed during this study are included in this article.
