# Supplementary material for: Circular RNA circ_IRAK3 contributes to tumor growth through upregulating KIF2A via adsorbing miR-603 in breast cancer
Source: Cancer Cell Int. 2022 Feb 14;22:81. doi: 10.1186/s12935-022-02497-y (PMC8845402; doi:10.1186/s12935-022-02497-y)
Supplement: Supplementary file 1 — Additional file 1: Figure S1. The experimental flowchart of this study. [file 12935_2022_2497_MOESM1_ESM.pptx]

## Slide 1
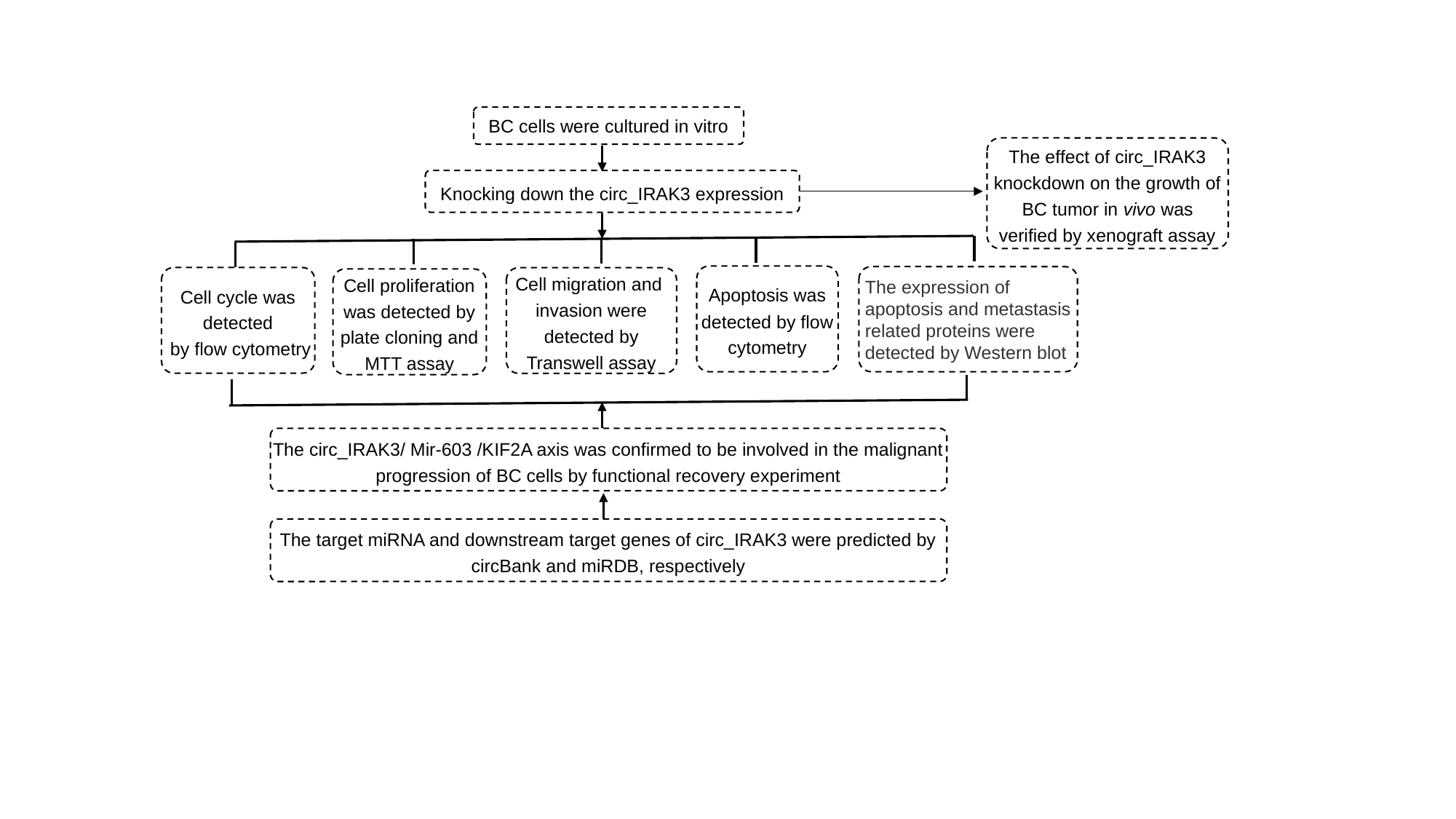

BC cells were cultured in vitro
The effect of circ_IRAK3 knockdown on the growth of BC tumor in vivo was verified by xenograft assay
Knocking down the circ_IRAK3 expression
Apoptosis was detected by flow cytometry
The expression of apoptosis and metastasis related proteins were detected by Western blot
Cell cycle was detected
 by flow cytometry
Cell migration and
invasion were detected by
Transwell assay
Cell proliferation was detected by plate cloning and MTT assay
The circ_IRAK3/ Mir-603 /KIF2A axis was confirmed to be involved in the malignant progression of BC cells by functional recovery experiment
The target miRNA and downstream target genes of circ_IRAK3 were predicted by circBank and miRDB, respectively
