# Supplementary figures and images for: Circular RNA circ_IRAK3 contributes to tumor growth through upregulating KIF2A via adsorbing miR-603 in breast cancer
Source: Cancer Cell Int. 2022 Feb 14;22:81. doi: 10.1186/s12935-022-02497-y (PMC8845402; doi:10.1186/s12935-022-02497-y)

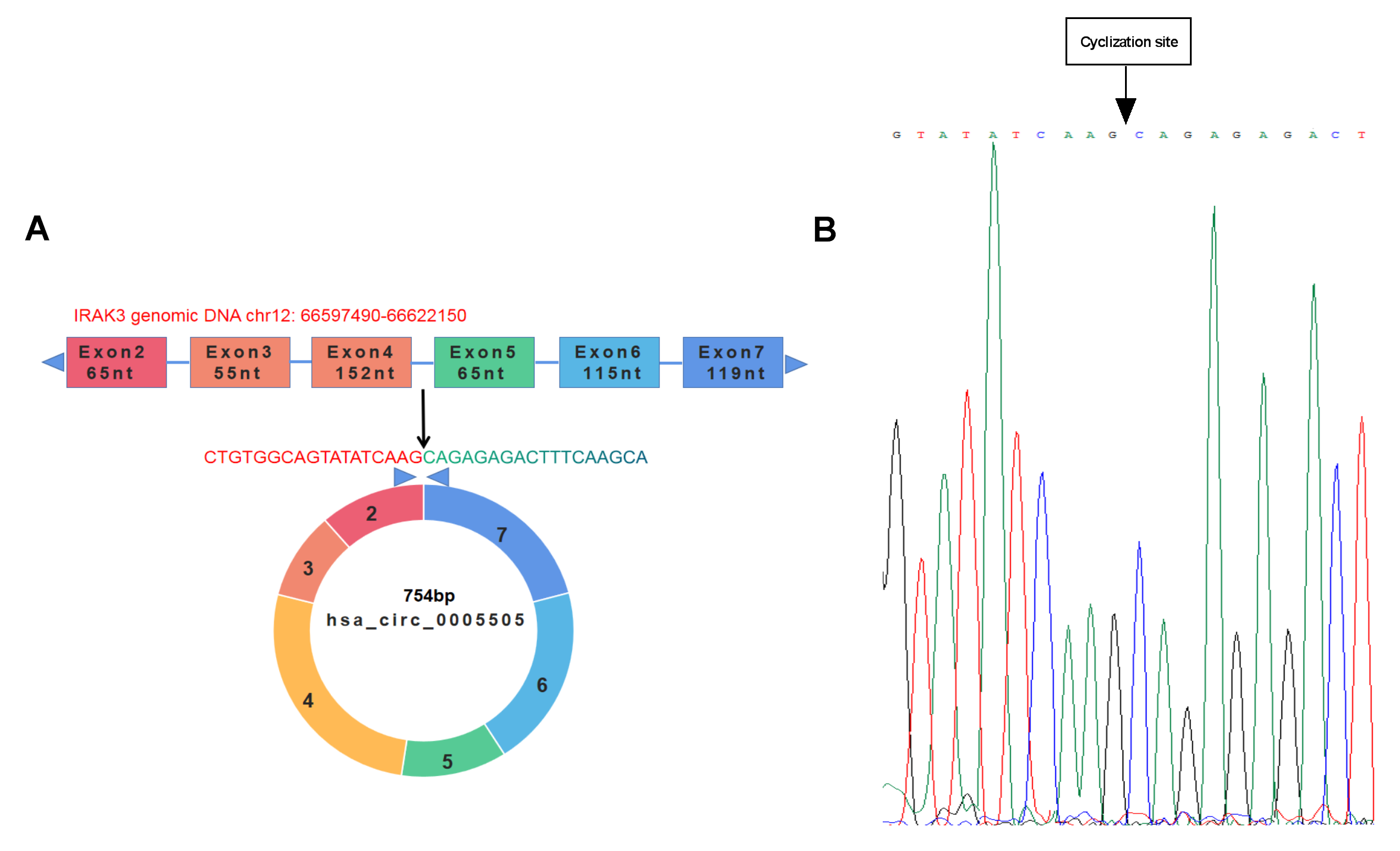

Supplement: Supplementary file 2 — Additional file 2: Figure S2. A A schematic diagram of circ_IRAK3 generated by the back-splicing of exons 2–7 of the IRAK3 gene. B Schematic diagram exhibiting a partial figure of the circ_IRAK3 sequencing result (the back-splicing region of circ_IRAK3). [file 12935_2022_2497_MOESM2_ESM.tif]

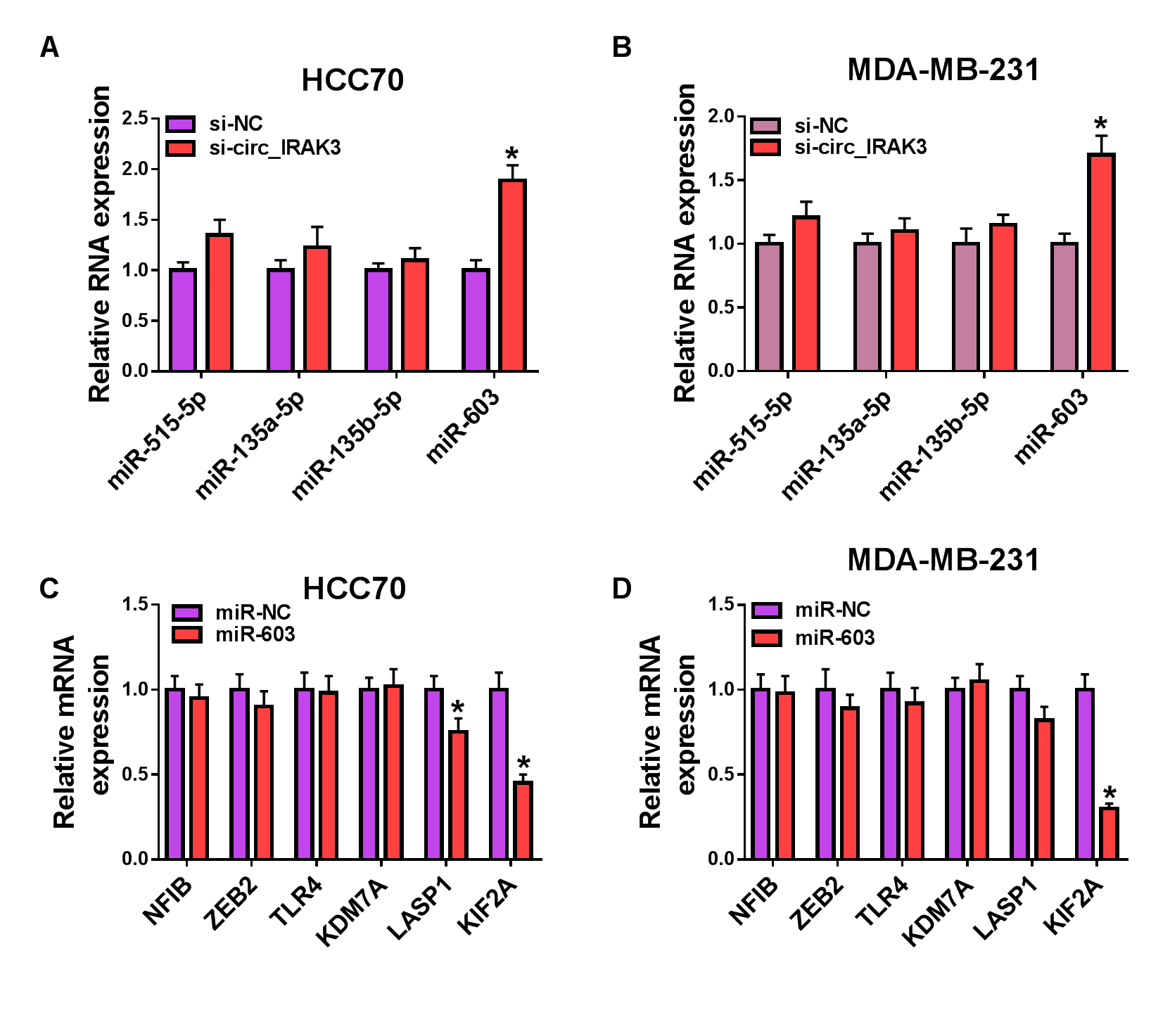

Supplement: Supplementary file 3 — Additional file 3: Figure S3. A, B QRT-PCR was performed to analyze the effect of Circ_IRAK3 silencing on the relative levels of 4 miRNAs (miR-515-5p, miR-135a-5p, miR-135b-5p, and miR-603). C, D QRT-PCR analysis of the effect of miR-603 overexpression on the relative levels of 6 candidate targets (NFIB, ZEB2, TLR4, KDM7A, LASP1, and KIF2A). *P < 0.05. [file 12935_2022_2497_MOESM3_ESM.tif]

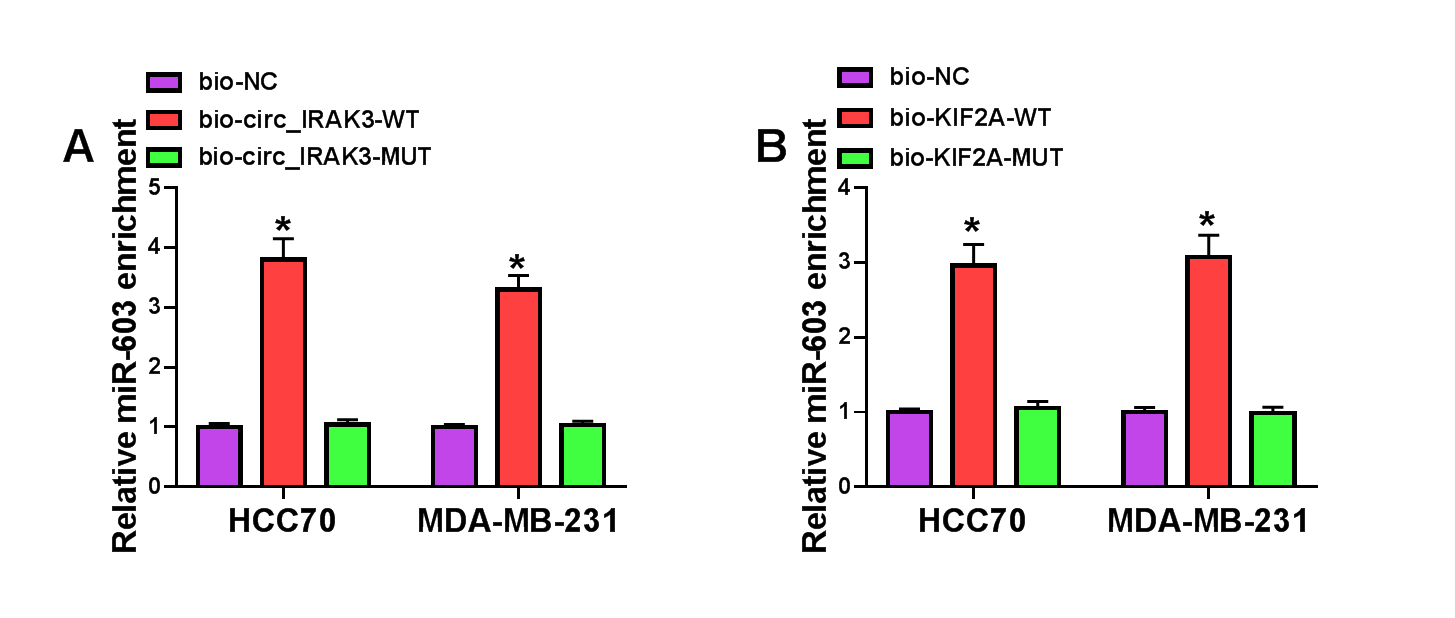

Supplement: Supplementary file 4 — Additional file 4: Figure S4. RNA pull-down assay analysis of the relationship between circ_IRAK3 or KIF2A and miR-603. *P < 0.05. [file 12935_2022_2497_MOESM4_ESM.tif]

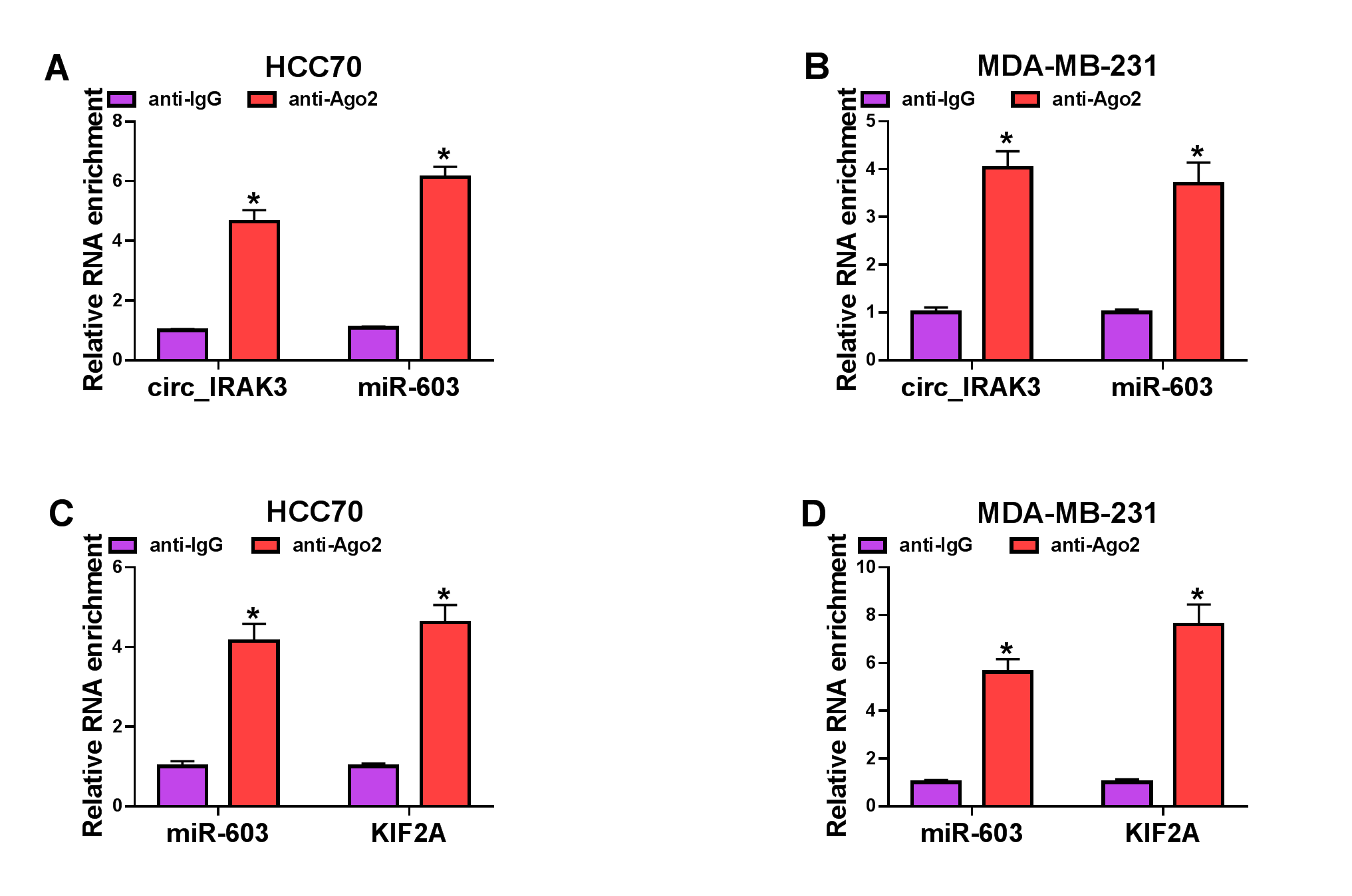

Supplement: Supplementary file 5 — Additional file 5: Figure S5. RIP assays showing the relationship circ_IRAK3 or KIF2A and miR-603. *P < 0.05. [file 12935_2022_2497_MOESM5_ESM.tif]

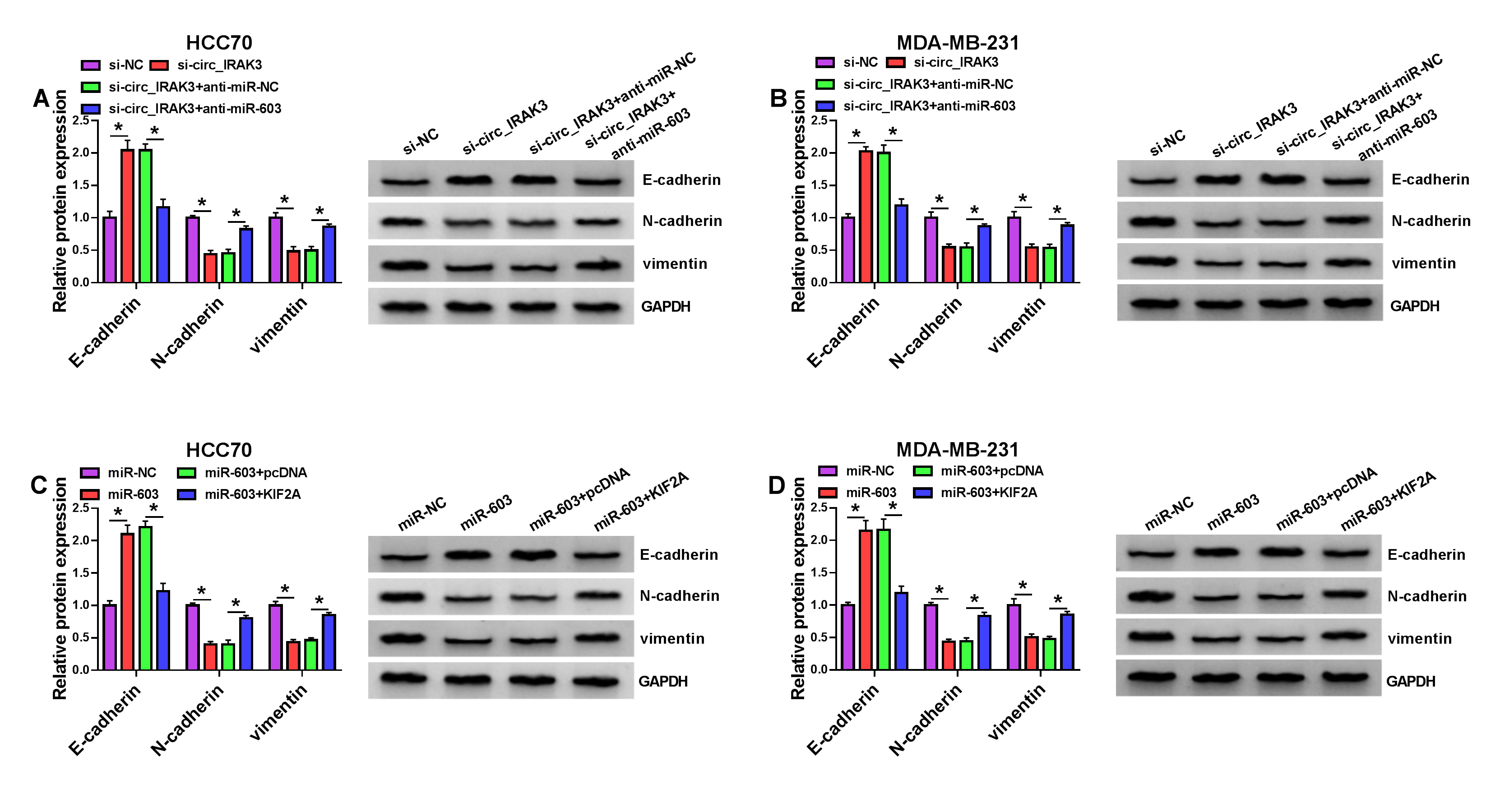

Supplement: Supplementary file 6 — Additional file 6: Figure S6. Western blotting exhibiting E-cadherin, N-cadherin, and vimentin protein levels in transfected HCC70 and MDA-MB-231 cells. *P < 0.05. [file 12935_2022_2497_MOESM6_ESM.tif]

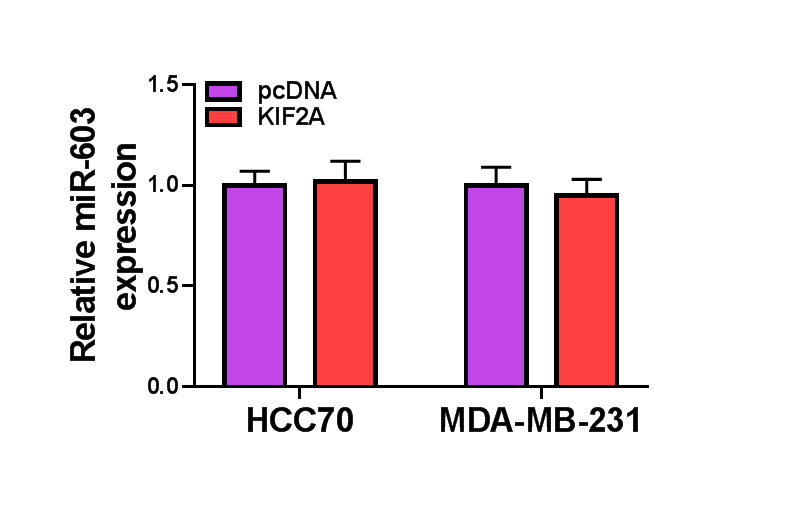

Supplement: Supplementary file 7 — Additional file 7: Figure S7. Effect of KIF2A overexpression on the expression of miR-603 in BC cells was determined by qRT-PCR. [file 12935_2022_2497_MOESM7_ESM.tif]

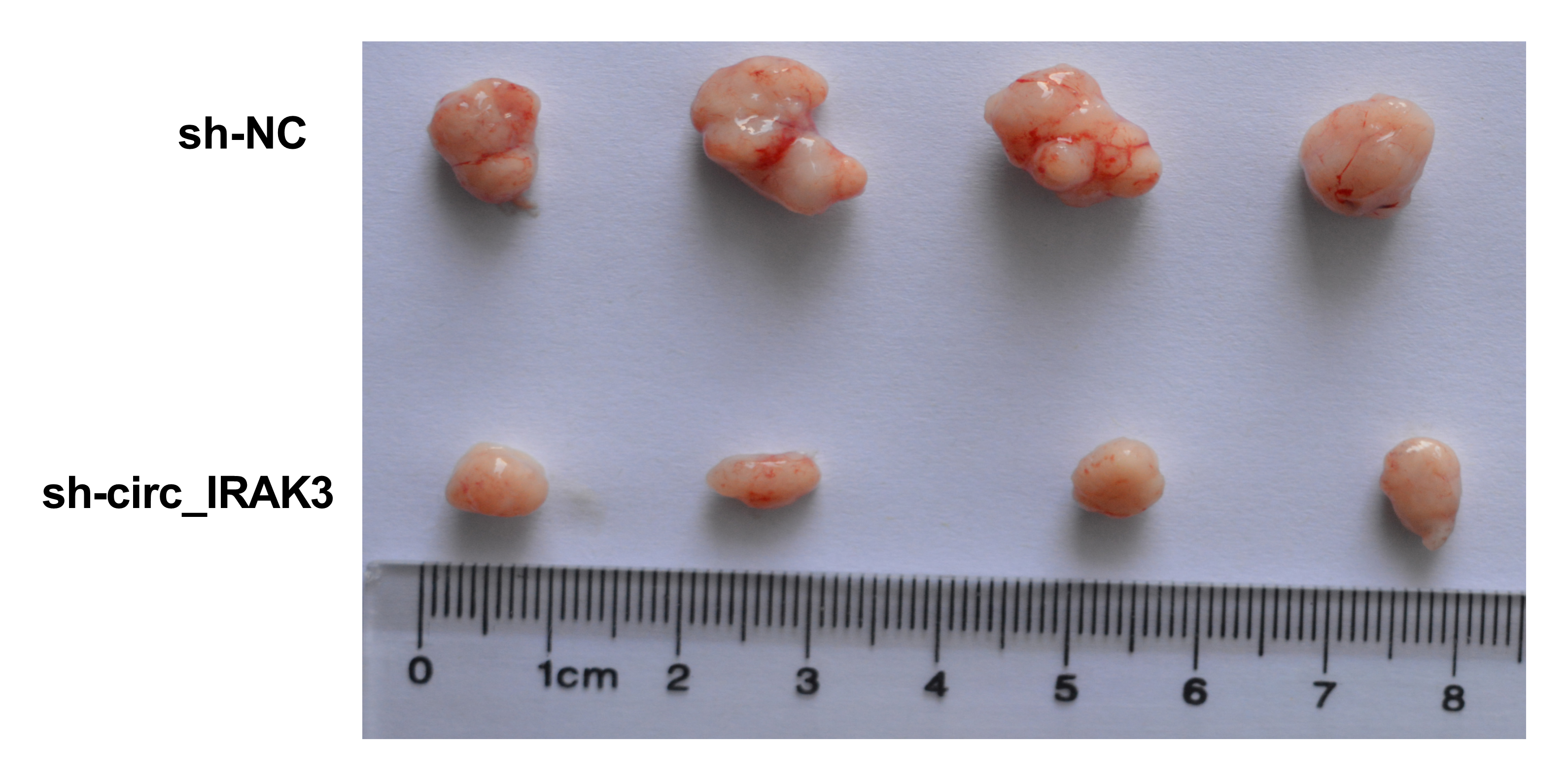

Supplement: Supplementary file 8 — Additional file 8: Figure S8. A picture showing other xenograft tumors not shown in Fig. 8. [file 12935_2022_2497_MOESM8_ESM.tif]
